# Toward PWAS: discovering pathways associated with human disorders

**DOI:** 10.1186/1471-2105-12-S11-A12

**Published:** 2011-11-21

**Authors:** Emre Guney, Baldo Oliva

**Affiliations:** 1 Structural Bioinformatics Group (GRIB-IMIM), Universitat Pompeu Fabra, Barcelona Research Park of Biomedicine (PRBB), 08003, Barcelona, Catalonia, Spain

## Introduction

The past decade has witnessed dramatic advances in genome sequencing and a substantial shift in the number of genome wide association studies (GWAS). These efforts have expanded considerably our knowledge on the sequential variations in Human DNA and their consequences on the human biology. Nevertheless, complex genetic disorders often involve products of multiple genes acting cooperatively and pinpointing the decisive elements of such disease pathways remains a challenge. Network biology recently proved its use in identifying candidate disease genes based on the simple observation that proteins translated by phenotypically related genes tend to interact, the so called guilt-by-association principle.

## Methods

Here, we present GUILD (Genes Underlying Inheritance Linked Disorders), a network-based candidate disease-gene prioritization framework which reveals the pathways associated with the disorder (pathway wide association study, PWAS). We exploit several distinct plausible communication mechanisms of known genes associated with the phenotype emerging from the topology of the interaction network. We used three sources of gene-phenotypic association to specify nodes involved in a disorder (seeds for the methods proposed): Online Mendelian Inheritance in Man (OMIM) database [1] and two published data sets (by Goh et al. [2], and Chen et al. [3]).

## Results and discussion

**Figure 1:**
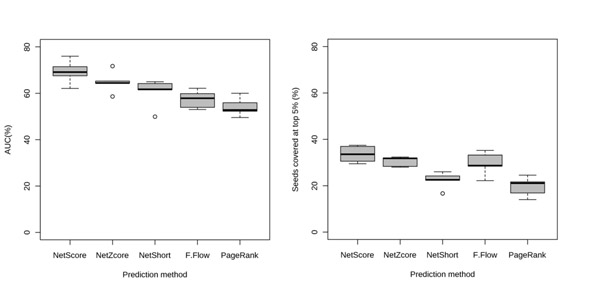
Comparison of prediction performance of proposed candidate disease-gene prioritization algorithms with existing methods. a) Area under ROC curve (AUC) yielded using 5 fold cross validation scheme over all disease-gene associations and different interaction sources used b) seed coverage among high scoring top 5% proteins.

Analysis on multiple human disease phenotypes demonstrate that the methods proposed in GUILD surpass state-of-the-art prioritization methods such as PageRank with priors [3] and Functional-Flow [4] (Fig. 1). We tested the robustness of the approaches proving the effect of the network properties and the independence with the number of original genes/proteins associated with the function or phenotype. We applied the prioritization methods to study the implication of pathways in various diseases and highlight the relationship between AD and aging. Our findings confirm that most prioritization methods introduced in this study are able to distinguish between groups of connected genes with functions identical to those of the known disease-associated genes (disease pathways). In addition, using prioritization methods, we increased the coverage of genes known to play important roles in the interplay of AD and aging, most of which would not be otherwise identified by just inspecting the direct neighborhood in the network.
